# Genome-Wide Analysis of the *Dof* Gene Family in Soybean and Functional Identification of *GmDof63* in Response to *Phytophthora sojae* Infection

**DOI:** 10.3390/plants14233621

**Published:** 2025-11-27

**Authors:** Sujie Fan, Haiyuan Chen, Yuhan Huo, Yang Song, Piwu Wang, Zhuo Zhang, Liangyu Jiang

**Affiliations:** Plant Biotechnology Center, College of Agronomy, Jilin Agriculture University, Changchun 130118, China; fansujie@jlau.edu.cn (S.F.); ch42@foxmail.com (H.C.); huoyuhan666@163.com (Y.H.); song-yang@jlau.edu.cn (Y.S.); peiwuw@163.com (P.W.)

**Keywords:** soybean, *Phytophthora sojae*, Dof transcription factors, genome-wide characterization, GmDof63

## Abstract

Phytophthora root and stem infection by *Phytophthora sojae* is a global and devastating disease of soybeans. Selecting disease-resistant varieties is the most economical and effective measure for controlling this disease. Delving into the disease resistance and defense molecular mechanisms can lay a theoretical foundation for solving this problem. Here, we screened the soybean genome and identified 78 *GmDof* genes distributed on nineteen chromosomes. Subcellular localization analysis revealed that the majority of GmDof proteins were located in the cell nucleus. Phylogenetic analysis categorized these genes into nine subfamilies. Gene structure analysis showed that all *GmDofs* contained 0 to 2 introns, and most of them did not have introns. Motif and conserved domain analysis showed that all GmDofs contained a common motif (motif-1) and a typical conserved C_2_-C_2_ domain. The prediction of *cis*-acting elements in promoter regions revealed numerous *cis*-regulatory elements responsible for stress responses, plant growth and development, plant hormone responses, and light responses. RNA-seq and quantitative real-time PCR results showed that *GmDof63* (Glyma.16G145000) was specifically expressed at high levels after *P. sojae* infection. *GmDof63* was strongly induced by SA and ETH treatments. The soybean seedlings overexpressing *GmDof63* displayed enhanced resistance to *P. sojae* infection compared with the wild-type soybean seedlings. Further experiments indicated that the expression levels of pathogenesis-related protein genes *PR1a*, *PR4*, *PR5a*, and *PR10* were significantly up-regulated in *GmDof63*-overexpressing transgenic soybean seedlings. Taken together, these findings reveal the mechanism by which *GmDof63* directly or indirectly regulates the expression of *PR* genes to modulate the soybean response to *P. sojae* infection.

## 1. Introduction

Soybean [*Glycine max* (L.) Merr.] is an important food and economic crop worldwide, and the significance and urgency of soybean research have become increasingly prominent [1,2]. Phytophthora root and stem rot of soybean is a devastating disease caused by *Phytophthora sojae*, leading to stunting, death or premature senescence of seedlings [3]. It can result in crop failure in the severely affected fields, causing billions of dollars of economic losses to the global soybean production each year [3,4]. Although several *P. sojae* strains and quantitative trait loci (QTLs) related to *P. sojae* resistance in soybean have been identified, the mechanisms underpinning the functions and regulation of resistance genes remain to be studied [5,6,7].

The DNA binding with one finger (Dof) family is a plant-specific class of transcription factors [8,9,10]. Members of the Dof family have been identified in many species, such as *Arabidopsis thaliana* [11], *Oryza sativa* [11], *G. max* [12], *Zea mays* [13], *Solanum tuberosum* [14], *Prunus persica* [15], and *Capsicum annuum* [16]. Dof transcription factors (TFs) typically consist of 200–400 amino acid residues and contain oligomerization sites [17]. The highly conserved domain consists of 50–52 residues at the N-terminus, containing a C_2_-C_2_ zinc finger domain CX_2_CX_21_CX_2_C [18,19]. Dof TFs recognize and bind to the AAAG-rich sequences or the CTTTT sequence of their target genes [20].

Dof TFs play a crucial role in various biological processes, including plant growth and development, signal transduction, and abiotic stress responses [21,22,23]. In Arabidopsis, the overexpression of *OBP4* (a Dof TF) promotes cell proliferation in the differentiation zone and induces the formation of callus [24]. In blueberry plants, *VcDof2* and *VcDof45* are considered to play significant roles in the flowering and fruit development processes, and *VcDof1*, *VcDof11* and *VcDof15* exhibit positive responses and upregulated expression under abiotic stress conditions [25]. The amount of oil contained in the seeds of cotton growing on specific land is related to *GhDof1*. When *GhDof1* is highly expressed, the oil content in cotton seeds will increase, while the protein content will decrease [26]. The cycling Dof factor 2 (CDF2) causes Arabidopsis to become insensitive to photoperiod and to delay flowering by reducing the level of *CONSTANS* [27]. In apple plants, MdCDOF3 and MdDOF3.6 activate the cytokinin oxidase *MdCKX7* in response to sorbitol signals, thereby accelerating the leaf senescence process [28]. The wheat endosperm-specific TF TaDOF6 promotes grain development by regulating the expression of *TaSWEET13h* and facilitating the transport of sugars and gibberellins [29]. SlDof22 can bind to the promoter of *SlSOS1* to down-regulate the expression of this gene, which compromises the salt stress tolerance [30]. In *Camellia sinensis*, *CsDOF51* and *CsDOF12* exhibit significant expression changes under drought stress and high-temperature stress, respectively, and *CsDOF44* shows significant changes under both conditions [31].

To date, the precise roles of Dof or Dof-like proteins in regulating biotic stress responses remain poorly understood. In tobacco, *Sar8.2b* can be activated by the Dof TF, which is related to systemic acquired resistance [32]. The atypical Dof TF OsDes1 can specifically recognize the promoter region of the defense-related gene *OsPR1b*, thereby activating the expression of *OsPR1b* to enhance the resistance to *Xanthomonas oryzae* pv. *oryzae* [33]. However, few studies report the *Dof* genes responsive to *P. sojae* infection in soybean. On another note, salicylic acid (SA), Methyl jasmonic acid (MeJA), ethylene (ETH), and abscisic acid (ABA) are all small molecule hormones, and they can coordinate the defense responses of plants through a complex signaling network, thereby regulating plant immunity [34,35,36,37]. Dof TFs have been shown to mediate responses to various hormone signals. *SsDOF1-7* in sugarcane have been shown to contain several *cis*-elements involved in SA, MeJA, and ABA responses [38]. In Arabidopsis, a DOF transcription factor gene, *OBP3*, is mainly responsive to SA, and DOF5.8 positively regulates the target gene *ANAC069* under abiotic stress conditions [39]. *FtDof* gene was mainly up-regulated under MeJA treatment but down-regulated under SA, ABA, gibberellic acid (GA), and indole-3-acetic acid (IAA) treatment in Tartary buckwheat [40]. The *VvDOF3* gene was rapidly induced by exogenous SA, MeJA, and powdery mildew infection [41]. These findings suggest that Dof TFs may play a key role by regulating the levels of plant hormone signals in response to environmental stress.

In this work, we sought to uncover the functional roles of the *Dof* gene family members in soybean defense against *P. sojae* infection. In order to do this, we identified 78 *Dof* family genes in soybean and analyzed their physicochemical properties, chromosomal localization, evolutionary relationships, gene structures, conserved motifs, intraspecies and interspecies collinearity, and promoter elements. We employed RNA sequencing and the quantitative RT-PCR method to screen out the key *Dof* gene in soybean response to *P. sojae* infection. Subsequently, *GmDof63* (Glyma.16G145000) was chosen for further analysis. Notably, *GmDof63* was significantly upregulated under SA and ETH treatments. The soybean seedlings overexpressing *GmDof63* displayed enhanced resistance to *P. sojae* infection compared with the wild-type (WT) soybean seedlings. These findings lay a good framework for further research into the involvement of the soybean *Dof* gene in defense against *P. sojae* infection.

## 2. Results

### 2.1. Identification and Characterization of the Dof Genes in Soybean

In this study, a total of 78 *Dof* genes were identified in the soybean genome and renamed as *GmDof1* to *GmDof78* based on the location order of soybean chromosomes from top to bottom. The GmDofs showed the amino acid sequence lengths ranging from 171 aa (GmDof18) to 503 aa (GmDof35), the molecular weights ranging from 19.65 kDa (GmDof18) to 54.73 kDa (GmDof35), and the theoretical pI ranging from 4.69 to 10.23, with 26 members having the pI less than 7 and 52 members having the pI greater than 7. Except for GmDof37, GmDof47, and GmDof60, the other members were unstable proteins. All the GmDofs were identified as hydrophilic proteins. Subcellular localization prediction indicated that except for GmDof47, located in the cytoplasm, and GmDof19 and GmDof60, located in the chloroplast, the other GmDofs were located in the cell nucleus (Table S1).

### 2.2. Chromosome Localization of GmDofs

**Figure 1:**
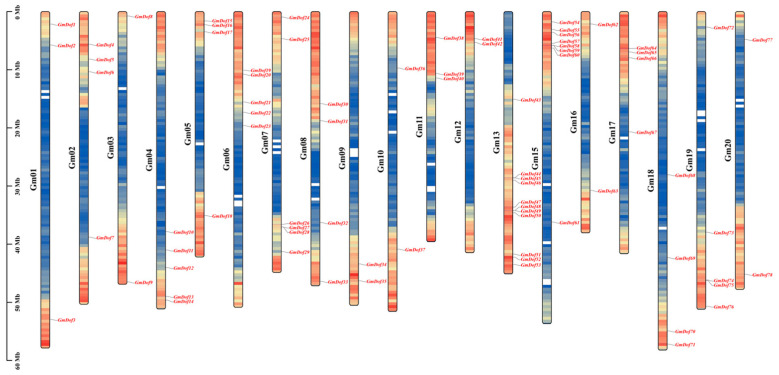
Genomic localization of soybean Dof members on chromosomes. Gene spacing are set at 200 kilobases, which is used to calculate the gene density on each chromosome. The color will gradually change from blue (low gene density) to red (high gene density) to represent this difference.

According to the soybean genome database, 78 soybean *Dof* genes were unevenly distributed among 20 chromosomes, with the exception of chromosome 14 (Figure 1). The number of *GmDofs* in each chromosome differed considerably. Chromosome 13 had the largest number (11) of *GmDofs*, followed by chromosome 15 (8) and chromosome 7 (6). Chromosomes 4, 6, and 19 had five *GmDofs*; chromosomes 2, 5, 8, 17, and 18 had four *GmDofs*; chromosomes 1 and 11 had three *GmDofs*; and chromosomes 3, 9, 10, 12, 16, and 20 had two *GmDofs*.

### 2.3. Phylogenetic Relationships of GmDofs

**Figure 2:**
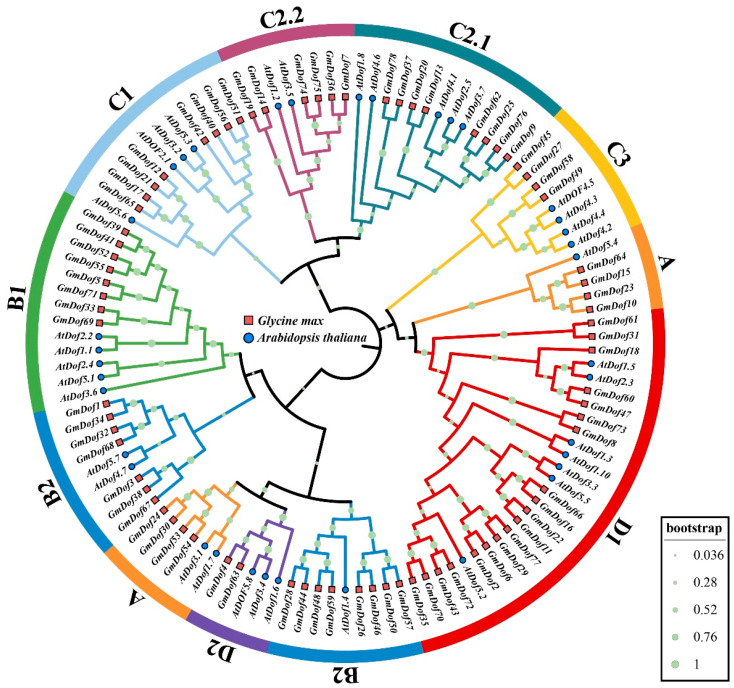
Phylogenetic tree of the Dof proteins in soybean and Arabidopsis.

To ensure the reliability of the analysis, we employed the Maximum likelihood (ML) method to perform multiple sequence alignment of GmDofs and 36 Arabidopsis Dof members and constructed a phylogenetic tree of all Dof proteins (Figure 2). The phylogenetic clustering of GmDofs was associated with the uneven distribution of their conserved domains. The GmDofs were clustered into nine subfamilies (A, B1, B2, C1, C2.1, C2.2, C3, D1, and D2), which showed significant differences in the number of GmDofs. Overall, the evolutionary tree structure of GmDofs is mainly related to the evolutionary relationships between species, and the genes of species with evolutionary relationships tend to cluster on the same branch of the evolutionary tree.

### 2.4. Gene Structures and Motif Distribution of GmDofs

**Figure 3:**
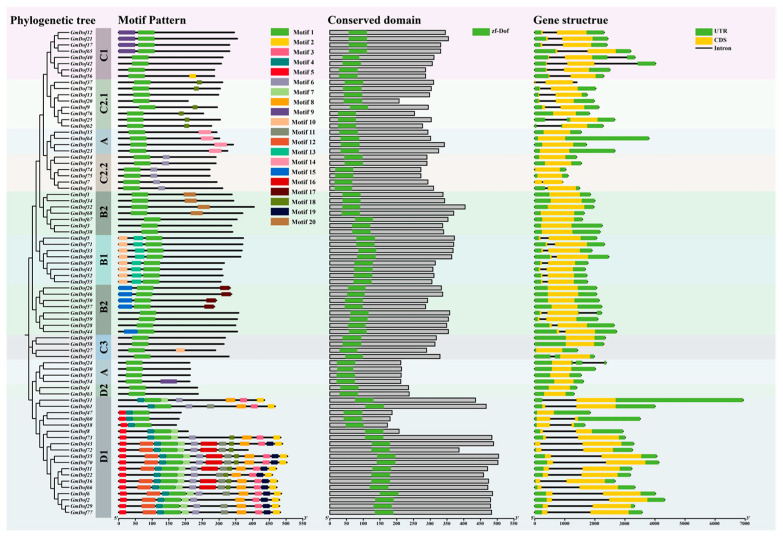
Visualization of motifs, domains, and gene structures of soybean Dof members.

To examine the structural diversity of GmDofs, we investigated the number of exon-introns and the distribution of conserved domains of GmDofs. This analysis provided evidence for unveiling the evolution of structural diversity in the soybean Dof family. Almost all *GmDofs* contained very few or no introns (Figure 3). The genes in the A, B2, and D2 subfamilies did not contain introns. The *GmDofs* classified in the same subfamily showed similar gene structures. To elucidate the evolution of GmDofs, MEME analysis identified 20 conserved motifs. Notably, N-terminal regions contained a highly conserved motif-1, and GmDofs within the same subfamily displayed similar motif composition (Figure 3). Most A subfamily members contained motif-1 and motif-14. B1 subfamily members contained motif-1, motif-10, and motif-13. Most B2 subfamily members contained motif-1, motif-17, and motif-20. Most C1 subfamily members contained motif-1 and motif-9. Most C2.1 subfamily members contained motif-1 and motif-18. C2.2 subfamily members contained motif-1 and motif-6. Most C3 subfamily members contained motif-1 and motif-10. Most D1 subfamily members contained motif-1, motif-2, motif-3, motif-4, motif-5, motif-6, motif-7, motif-8, motif-11, motif-12, motif-16, motif-18, and motif-19. D2 subfamily members only contained motif-1. All GmDofs contained the typical conserved Dof domain, categorized under the zinc finger superfamily, indicating evolutionary conservation of functional domains among GmDofs.

### 2.5. Collinearity of GmDofs

**Figure 4:**
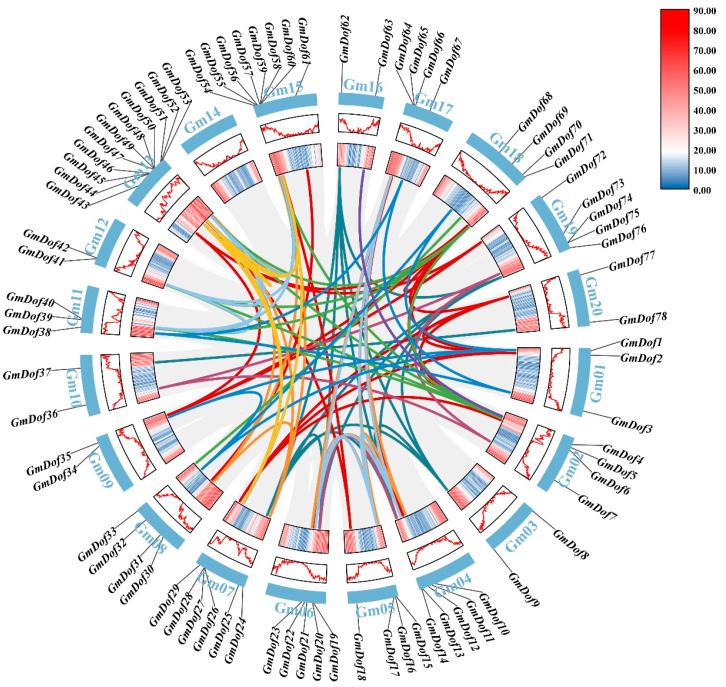
Synteny analysis of interchromosomal relationships of soybean *Dof* members. This curved structure represents chromosomes, and the different colored lines indicate segmental gene pairs.

To further elucidate the potential functions of the *GmDofs*, we analyzed the duplication events giving rise to *GmDofs*. The distribution of *GmDofs* varied among different chromosomes, indicating substantial differences in their chromosomal evolution. The soybean genome carries 110 *GmDof* gene pairs involved in duplication events, which are located on different chromosomes, with the greatest number of *GmDof* homologous gene pairs on chromosome 13 (Figure 4). The findings indicate that gene segmental duplication events may have been the main driving force behind the evolution of *GmDofs*. Furthermore, we calculated the synonymous (Ks) and non-synonymous (Ka) substitution rates (Ka/Ks) of 110 segmentally duplicated pairs (Table S2). The Ka/Ks ratios for segmentally duplicated gene pairs ranged from 0.07 to 0.64, with an average of 0.29, indicating that the *GmDofs* have undergone strong negative purifying selection pressure. Furthermore, the frequency distribution of the Ka/Ks ratios showed that more than 78% of duplicated gene pairs had ratios ranging from 0.1 and 0.3. These results demonstrate the conserved evolution of soybean *Dof* genes.

### 2.6. Interspecies Collinearity of GmDofs

**Figure 5:**
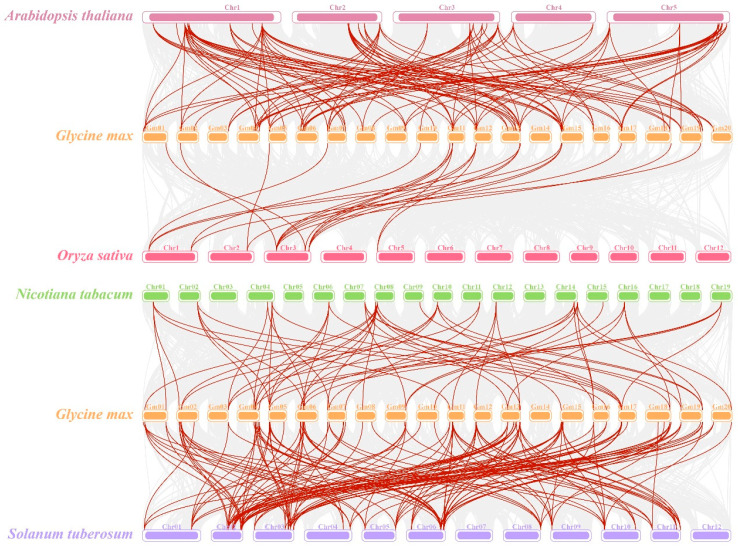
Synteny analysis of *Dof* genes. Each horizontal line represents a chromosome, and the red lines indicate the *Dof* homologous gene pairs.

To further investigate the gene duplications in the *Dof* genes, we analyzed the collinearity of *GmDofs* with the *Dof* genes in other species, including *A. thaliana*, *O. sativa*, *Nicotiana tabacum*, and *S. tuberosum* (Figure 5). The results showed that the collinear gene pairs in rice, a monocot model plant, were significantly fewer than those in dicot genomes. In addition, more genetic overlap was found between soybean and *S. tuberosum* than between soybean and other plants, signifying a closer evolutionary relationship of soybean to *S. tuberosum*.

### 2.7. Predicted Cis-Acting Elements in the Promoters of GmDofs

**Figure 6:**
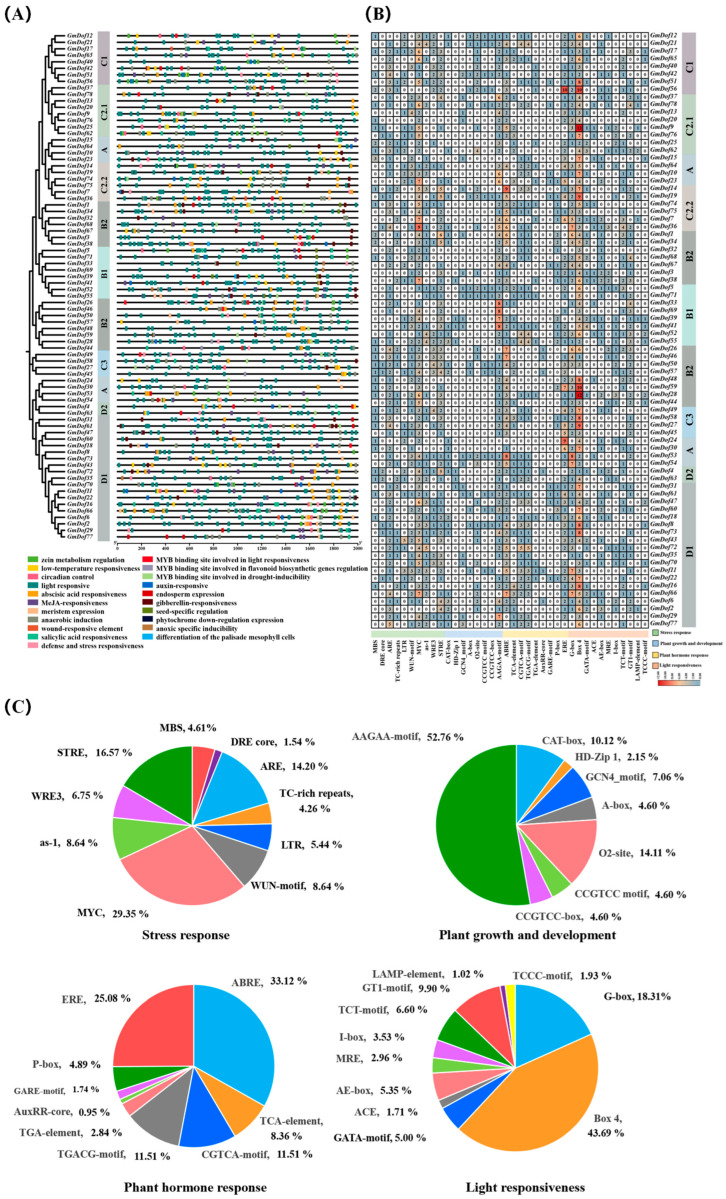
*Cis*-acting elements in the promoters of soybean *Dof* members. (**A**) Variations in different types of *cis*-acting elements. (**B**) Number of *cis*-acting elements. (**C**) Pie charts show the proportion of different *cis*-acting elements in each category.

In this study, PlantCARE was used to analyze the *cis*-acting elements in the sequence upstream (2 kb) from the start codon of each *GmDof*. The results showed that the responsive elements were widely present in the *Dof* family genes of soybean (Figure 6A). All *cis*-acting elements were classified into four categories according to their functions: stress response, plant growth and development, plant hormone response, and light response (Figure 6B,C). Stress response-related element analysis showed that MYC, a drought-responsive element, was the most numerous (29.35%), and STRE (a stress-responsive element) was the second most numerous element (16.57%). The promoters of 92.30% of *GmDofs* contained the MYC element, and those of 78.21% of *GmDofs* contained the STRE element, suggesting that *GmDofs* played a role in regulating drought and stress responses. Plant growth and development-related element analysis showed that the AAGAA motif, an auxin-responsive element, was the most numerous (52.76%). The promoters of 87.18% of *GmDofs* contained the AAGAA motif, suggesting that most *GmDofs* played a role in regulating auxin responses. The analysis of plant hormone response-related elements showed that ABRE was the most numerous element (33.12%), which was the ABA-responsive element. The promoters of 83.33% of *GmDofs* contained the ABRE element, suggesting that *GmDofs* played an important role in regulating ABA response. Among the light-responsive elements, the box 4 element was the most numerous (43.69%). The promoters of 97.44% of *GmDofs* contained the box 4 element, suggesting that *GmDofs* played an important role in light response.

### 2.8. Screening of Dof Genes in Soybean After P. sojae Infection

**Figure 7:**
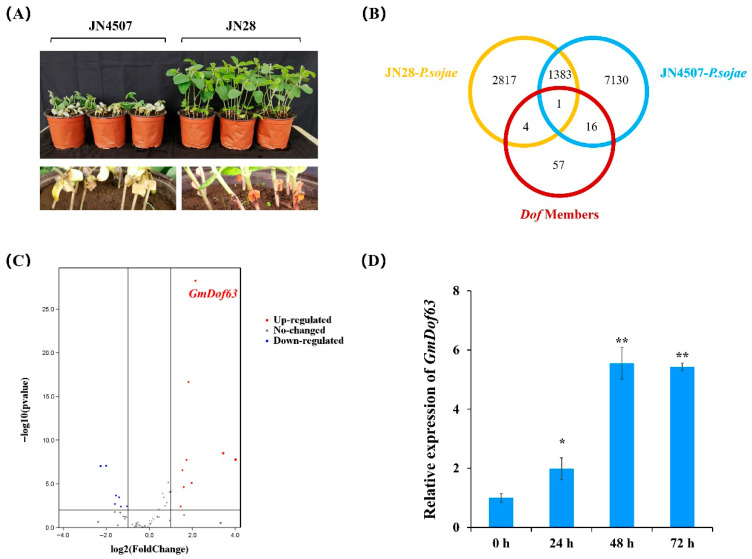
Screening of *Dof* genes in soybean after *P. sojae* infection. (**A**) The phenotypic characteristics of the resistant variety and the susceptible variety after *P. sojae* infection. (**B**) Overlapping between JN28-*P. sojae*, JN4507-*P. sojae* and *Dof* genes. (**C**) Volcano plot of soybean Dof members. (**D**) Expression levels of *GmDof63* in JN28 seedlings during *P. sojae* infection. Three biological replicates were used for each sample, and Student’s *t*-test (* *p* < 0.05, ** *p* < 0.01) was conducted to determine statistical significance. Error bars represent ±SD.

Hypocotyls of JN4507 and JN28 seedlings were inoculated with *P. sojae* isolate PSR01. At 48 h post-inoculation of *P. sojae*, it was observed that the JN28 soybean seedlings remained firm, with only slight browning of the stems, while the JN4507 soybean seedlings exhibited extended lesions, with the entire plant wilting and emitting a foul odor (Figure 7A). The seedling incubation assay demonstrated that JN28 showed a strong immune response against *P. sojae* isolate PSR01, whereas JN4507 was completely susceptible.

To determine whether Dof members control *P. sojae* infection, we profiled the expression of *GmDofs* in soybean seedlings during *P. sojae* infection by RNA sequencing. We collected three replicates of *P. sojae*-treated JN28 and JN4507 samples at 48 h post-inoculation to capture the transcriptional changes during *P. sojae* infection. The control samples were inoculated with an agar block without *P. sojae*. The differentially expressed genes were identified by a pairwise comparison of the transcriptome datasets (JN28-*P.* sojae vs. JN4507-P. *sojae*) (Figure 7B). We further identified the significantly upregulated expression of *GmDof63* (Glyma.13G329000) through the volcano plot (Figure 7C). Quantitative RT-PCR results indicated that the expression of *GmDof63* was upregulated in the highly resistant soybean variety JN28 after *P. sojae* infection (*p* < 0.01), and the accumulation of *GmDof63* peaked at 48 h post-inoculation (Figure 7D). These results indicated that *GmDof63* was a potentially crucial gene in soybean during *P. sojae* infection.

### 2.9. Sequence Characteristics and Expression Pattern of GmDof63

**Figure 8:**
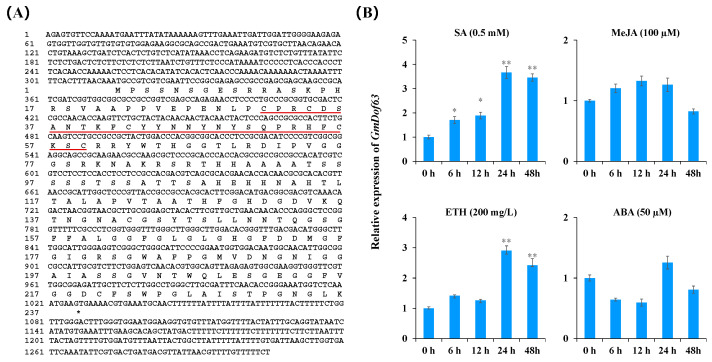
Sequence characteristics and expression pattern of *GmDof63*. (**A**) The *GmDof63* gene sequence and the GmDof63 protein sequence. The zinc finger domain CX_2_CX_21_CX_2_C are underlined in red. (**B**) Expression pattern of *GmDof63* in soybean under different stresses. The various treatments were the 0.5 mM SA treatment, 100 µM MeJA treatment, 200 mg/L ETH treatment and 50 µM ABA treatment. Three biological replicates were used for each sample, and Student’s *t*-test (* *p* < 0.05, ** *p* < 0.01) was performed to determine statistical significance. Error bars represent ±SD.

To study *GmDof63* expression, we first cloned the complete sequence of *GmDof63* from JN28 using the RT-PCR technique, which contained a 711 bp open reading frame that encodes a 236-amino acid protein with the zinc finger domain CX_2_CX_21_CX_2_C (Figure 8A). We further explored the expression pattern of *GmDof63* in soybean under different stresses. As shown in Figure 8B, the expression of *GmDof63* was significantly upregulated at 6 h and reached a peak at 24 h after SA treatment, and the expression of *GmDof63* was significantly upregulated at 24 h and reached a peak at 24 h after ETH treatment, but the expression level of the *GmDof63* did not show any significant change after treatment with MeJA and ABA. The results suggested that *GmDof63* participates in multiple signaling pathways.

### 2.10. Subcellular Localization of GmDof63

**Figure 9:**
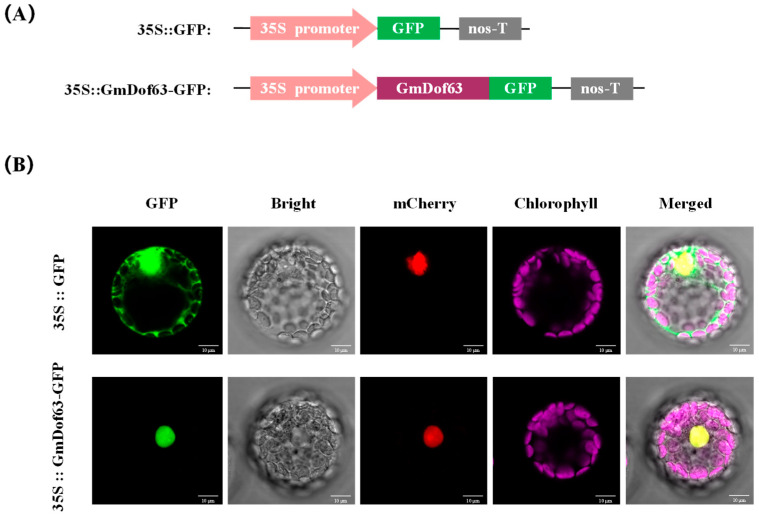
Subcellular location of GmDof63 in the Arabidopsis mesophyll protoplasts. (**A**) Construction of 35S::GFP vector and 35S::GmDof63-GFP vector. (**B**) 35S::GFP vector or 35S::GmDof63-GFP vector was transiently expressed in the Arabidopsis mesophyll protoplasts, respectively. The green fluorescence signal indicates the 35S::GmDof63-GFP or 35S::GFP protein, and the red fluorescence signal indicates the nuclear marker protein H2B-mCherry, and the fluorescence signals were observed under a confocal microscope. Scale bars = 10 µm.

To investigate the subcellular localization of GmDof63, 35S::GFP vector or 35S::GmDof63-GFP vector (Figure 9A) was transformed into Arabidopsis mesophyll protoplasts. H2B-mCherry was chosen as the nuclear marker protein, which can encode histone H2B fused with the red fluorescent protein mCherry. As can be seen from Figure 9B, the transformed cells carrying 35S::GFP showed a strong green fluorescence signal throughout the entire cell, whereas the transformed cells carrying 35S::GmDof63-GFP showed a strong green fluorescence signal only in the nucleus, and this finding was consistent with the location of the nuclear marker protein H2B-mCherry. These results suggested that GmDof63 is a nucleus-localized transcription factor.

### 2.11. GmDof63 Enhances Resistance of Transgenic Soybean Seedlings to P. sojae

**Figure 10:**
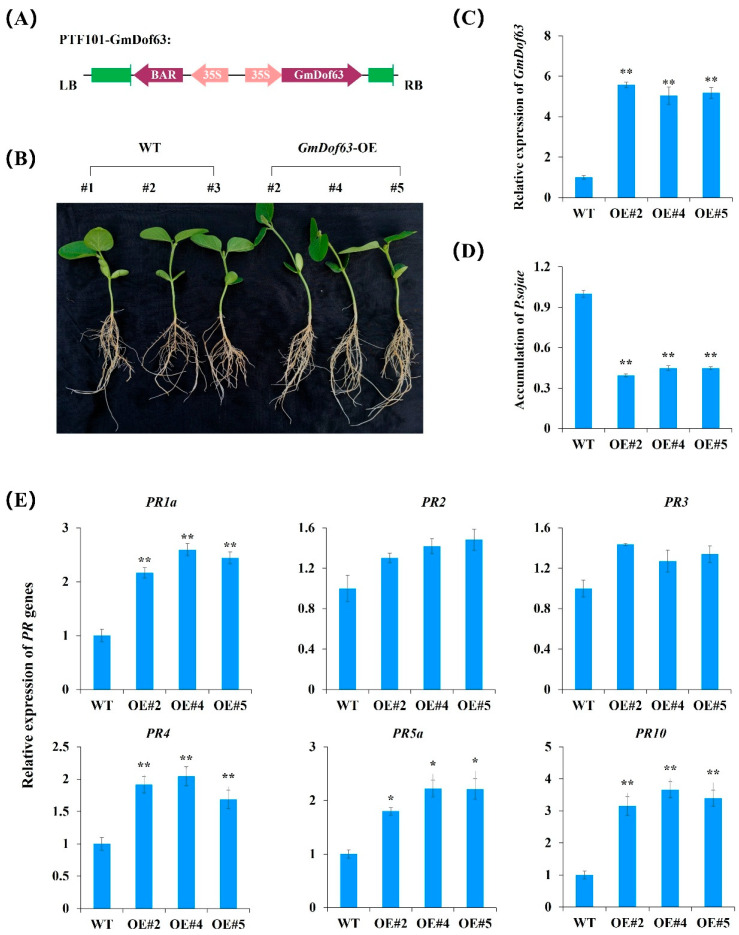
*GmDof63* enhances soybean resistance to *P. sojae*. (**A**) Construction of *GmDof63*-overexpressing vector 35S::*GmDof63*. (**B**) Phenotypes of WT and *GmDof63*-overexpressing transgenic soybean seedlings treated with *P. sojae* for 48 h in the soybean root hydroponic assay. (**C**) Relative expression levels of *GmDof63* in WT and *GmDof63*-overexpressing transgenic soybean seedlings at 48 h post-inoculation. (**D**) Accumulation of *P. sojae* in WT and *GmDof63*-overexpressing transgenic soybean seedlings at 48 h post-inoculation. The relative accumulation of *P. sojae* in roots was measured based on the relative expression of *P. sojae* housekeeping gene *PsACT* (XM_009530461.1) to soybean housekeeping gene *GmActin* (Glyma.18G290800.1) (ΔCt = Ct*_HK_*_of *P. sojae*_ − Ct*_HK_*_of soybean_). (**E**) Relative expression levels of *PR1a* (AF136636), *PR2* (M37753), *PR3* (AF202731), *PR4* (BT090788), *PR5a* (M21297), *PR10* (FJ960440) in *GmDof63*-overexpressing transgenic soybean seedlings were compared with those in WT. WT, wild type. Three biological replicates were used for each sample, and Student’s *t*-test (* *p* < 0.05, ** *p* < 0.01) was performed to determine statistical significance. Error bars represent ±SD.

We next focused on the function of *GmDof63*. To confirm the functions of *GmDof63* in soybean response to *P. sojae*, we constructed a *GmDof63*-overexpressing vector 35S::*GmDof63* with BAR as the selective marker (Figure 10A). We then used an efficient *Agrobacterium*-mediated transformation system described by Paz et al. (2004) [42] and Li et al. (2017) [43] to generate *GmDof63*-overexpressing transgenic soybean seedlings. In the T0 and T2 generations, the transgenic soybean seedlings were verified by the BAR LibertyLink strip (Envirologix, Portland, OR, USA) (Figure S1). Three lines of positive transgenic soybean seedlings were inoculated with *P. sojae* zoospores in a hydroponic assay. The zoospore suspension was prepared according to the method described by Shrestha et al. (2016) [44] and Yang et al. (2021) [45]. Under standard culture conditions, no significant differences in leaf phenotype were observed between transgenic and WT plants. However, the WT soybean roots exhibited extended lesions, browning, and wilting, while the *GmDof63*-overexpressing soybean roots remained firm with only slight browning (Figure 10B). Quantitative RT-PCR results showed that the expression of *GmDof63* in *GmDof63*-overexpressing soybean roots was higher than that of WT soybean roots at 48 h post-inoculation (Figure 10C). The relative accumulation of *P. sojae* was significantly lower in *GmDof63*-overexpressing soybean roots than in WT soybean roots at 48 h post-inoculation (Figure 10D). The above results indicated that the *GmDof63*-overexpressing transgenic soybean seedlings displayed enhanced resistance to *P. sojae* infection compared with the WT soybean seedlings.

Pathogenesis-related (*PR*) genes are some of the most important genes in the defense response of plants against pathogens [46,47,48,49]. To test whether GmDof63 can regulate the expression of *PR* genes, we performed quantitative RT-PCR analysis in roots of *GmDof63*-overexpressing and WT soybean at 48 h post-inoculation. The expression of *PR1a*, *PR4*, *PR5a*, or *PR10* in *GmDof63*-overexpressing soybean roots was higher than that of WT soybean roots (Figure 10E). Previous research has revealed that Dof proteins often recognize the AAAG or TTTC motif in their target promoters [50,51,52], so we analyzed the AAAG or TTTC motif in the promoter sequence of the *PR* genes. The result showed that a large number of AAAG or TTTC motifs were present in the promoter sequence of *PR1a*, *PR4*, *PR5a*, and *PR10* (Figure S2), indicating that *GmDof63* may directly or indirectly regulate the expression of *PR* genes to modulate the soybean response to *P. sojae* infection.

## 3. Discussion

The Dof family is a type of plant-specific TFs belonging to the single zinc finger protein superfamily. With the development of bioinformatics, since the first *Dof* gene was cloned from maize, the *Dof* gene family has been deeply identified and analyzed in plants such as Arabidopsis, rice, maize, potato and sweet pepper [23,53]. However, the research on *Dof* genes in soybean is still limited, and there are few reports on the roles of *Dof* genes in response to *P. sojae* infection.

In this study, we identified 78 members of the Dof family in soybean and analyzed their physicochemical properties, chromosomal localization, evolutionary relationships, gene structures, intraspecies and interspecies collinearity, and promoter elements. Subcellular localization is an important characteristic of proteins, and protein function is closely related to protein localization [54,55]. For example, proteins in the cell nucleus mainly participate in gene transcription and DNA repair [56]. In this study, the subcellular localization prediction showed that 75, 1, and 2 GmDofs were located in the cell nucleus, cytoplasm, and chloroplast, respectively.

Motif, conserved domain, and gene structure prediction indicated that all GmDofs contained a common motif (motif-1), suggesting that this motif may play a key role. The diversity of motifs among different Dof members may be related to their complex functions. Different gene families have their own conserved domains. All GmDofs had a complete C_2_-C_2_ single-finger zinc structure, which was consistent with the research results of Wu et al. (2019) [57] and Luo et al. (2022) [58]. Gene structure analysis showed that all *GmDofs* had 0 to 2 introns, and most of them did not have introns. This feature indicated that the gene structure of *GmDofs* was conserved.

The evolution and expansion of gene families are closely related to gene duplication. The occurrence of gene duplication may be due to fragment duplication, tandem duplication or whole-genome duplication [59,60]. Some duplicated genes may retain similar functions and show partial or complete divergence from each other [61]. Many TF families in plants have undergone gene duplication events [62]. In this study, we discovered that *GmDofs* were unevenly distributed on chromosomes other than chromosome 14. Homology analysis showed that the soybean genome contained 110 pairs of *Dof* homologous genes, all of which originated from fragment duplication, indicating that fragment replication may have played a dominant role in the evolution and expansion of the *GmDof* family. In addition, the Ka/Ks values of 110 pairs of *Dof* homologous genes were all less than 1, indicating that the *Dof* genes have undergone purifying selection during long-term evolution. Interspecies collinearity showed that the number of co-linear *Dof* gene pairs of soybean with monocotyledonous model plants such as rice was significantly lower than that with dicotyledonous plants such as *A. thaliana*, *O. sativa*, *N. tabacum* and *S. tuberosum*, and the gene overlap with *S. tuberosum* was the most, indicating a closer evolutionary relationship with *S. tuberosum*.

Dof TFs play a significant role in regulating plant growth and development, stress responses, and plant hormone signal transduction [63,64,65,66,67]. Studies have shown that *cis*-acting elements are involved in the responses to various environmental stresses [68,69,70,71]. The MYC and STRE elements play an important role in stress responses. The AAGAA-motif element is involved in plant responses to environmental stresses. ABRE played an important role in regulating ABA response. The Box 4 element acts as a photosensitive element, participating in the plant response to light conditions. We can further understand the functions of *GmDofs* by analyzing the *cis*-acting elements of the promoters of *GmDofs*. The most common *cis*-acting elements predicted in the promoter region of *GmDofs* contained MYC, STRE, AAGAA-motif, ABRE, and Box 4 elements. Therefore, *GmDofs* play a crucial role in plant growth and development, stress responses, and plant hormone signal transduction.

Phytophthora root and stem rot, a devastating disease caused by *P. sojae*, has caused serious losses to soybean production worldwide. Studies have confirmed that there are extensive genetic variations (including those conferring disease resistance) in major crops, providing an opportunity to utilize these variations to enhance the soybean response to *P. sojae*. For example, miR393 and miR166 have been found to play a crucial role in responding to *P. sojae* infection [72]. Overexpression of the bHLH transcription factor *GmPIB1* can enhance resistance to *P. sojae* [73]. The zinc finger protein-type TF GmZFP03 boosts the resistance to *P. sojae* by targeting the promoters of two *SOD1* genes and activating their expression [74]. GmCAT1 can slightly cause cell death in Arabidopsis and promote the development of *P. sojae*, and the interaction between PsAvh113-GmDPB-GmCAT1 may be a potential defense mechanism that is conducive to the infection of *P. sojae* [75]. However, the natural variations in *Dof* genes have not been examined in soybeans responding to *P. sojae* infection. Here, we identified one *Dof* gene, *GmDof63*, in soybean via RNA sequencing, which exhibited an upregulated expression in the resistant materials. The function of *GmDof63* was verified through genetic transformation. The *GmDof63*-overexpressing transgenic soybean seedlings demonstrate enhanced resistance to *P. sojae*. Quantitative RT-PCR results showed that the expression level of *GmDof63* significantly increased in *GmDof63*-overexpressing transgenic soybean after *P. sojae* infection, and the relative accumulation of *P. sojae* was significantly lower in *GmDof63*-overexpressing transgenic soybean than in WT soybean, further indicating that this gene played a role in the response to *P. sojae* infection.

Much research has shown that Dof TFs play a key role in mediating plant hormone pathways [38,39,40,41,76]. We discovered the similar research result that the transcription level of *GmDof63* in soybean seedlings was significantly induced after spraying SA and ETH. Previous studies have shown that transcription factors can specifically bind to the promoters of *PR* genes to regulate their expression, thereby responding to pathogen infection [77,78]. In this study, the expression levels of *PR1a*, *PR4*, *PR5a* and *PR10* were significantly up-regulated in *GmDof63*-overexpressing transgenic soybean seedlings, and a large number of AAAG or TTTC motifs were present in the promoter sequence of *PR1a*, *PR4*, *PR5a*, and *PR10*, so we speculated that *GmDof63* may directly or indirectly regulate the expression of these *PR* genes. Furthermore, previous research has also revealed that *PRs* usually function as effector genes for systemic acquired resistance (SAR), and this resistance is mediated by SA [79,80]. So, we speculate that the high expression levels of *PR* genes indicate the activation of the SA signaling pathway. Taken together, these findings suggested that *GmDof63* may serve as a key regulatory center for the SA and ETH signaling pathways, integrating these pathways to cope with *P. sojae* infection.

In the follow-up work, the phenotypes of *GmDof63*-overexpressing transgenic soybean and the expression levels of *GmDof63* and *PRs* will be evaluated at multiple time points after *P. sojae* infection, which could offer a more detailed understanding of the temporal dynamics of the defense response. Now, the functional verification of *GmDofs* in response to *P. sojae* infection can not only reveal the molecular mechanism underpinning the soybean response to pathogen infection but also provide direct molecular markers and candidate genes for the breeding of new varieties with specific disease resistance traits, demonstrating promising application prospects.

## 4. Materials and Methods

### 4.1. Identification of Dof Members in Soybean Genome

The soybean Wm82.a4.v1 genome was downloaded from the Soybase database (https://www.soybase.org/ (accessed on 12 April 2023)) for the identification of *Dof* genes. The nucleotide and protein sequences of Dof family members of *A*. *thaliana* were downloaded from TAIR (https://www.arabidopsis.org/ (accessed on 12 April 2023)), and the nucleotide and protein sequences of Dof family members of *O*. *sativa* were downloaded from the Rice Genome Annotation Project (http://rice.uga.edu/ (accessed on 12 April 2023)). The Hidden Markov Model file corresponding to the Dof domain (PF02701) was downloaded from the Pfam database (http://pfam.xfam.org/ (accessed on 13 April 2023)). Dof members were searched from the soybean database via HMMER 3.0 (http://hmmer.org/ (accessed on 15 April 2023)). The BLASTp v2.12.0 tool was used to compare these sequences against the acquired Dof protein sequences of *O*. *sativa* and *A. thaliana*, and the genes with E-values ≤ 1 × 10^−5^ were retained. These collected putative Dof members were confirmed by the Pfam (http://pfam.sanger.ac.uk/ (accessed on 15 April 2023)) and InterPro (https://www.ebi.ac.uk/interpro/ (accessed on 15 April 2023)) databases. The longest CDS transcript in the same gene was selected as the representative sequence.

### 4.2. Physicochemical Characterization of Soybean Dof Members

The physicochemical properties of the predicted proteins of soybean *Dof* genes were calculated via the ProtParam tool (https://web.expasy.org/protparam/ (accessed on 26 April 2023)), including amino acid sequence length, theoretical isoelectric point (pI), molecular weight, instability index, aliphatic index, and grand average of hydropathicity. The subcellular localization of the soybean Dof members was predicted by Wolf Psort online (https://wolfpsort.hgc.jp/ (accessed on 18 May 2023)), and the top predicted position was selected as the result.

### 4.3. Chromosomal Location Analysis of Soybean Dof Members

Gene Location Visualize from GTF/GFF module in Tbtools v2.310 software was used to map all non-redundant soybean *Dof* genes on the 20 soybean chromosomes on the basis of the information in the soybean database.

### 4.4. Phylogenetic Analysis of Soybean Dof Members

The amino acid sequences of Dof members from soybean and Arabidopsis were selected for phylogenetic analysis. ClustalW and ClustalX with default parameters were used for multiple sequence alignment. The phylogenetic tree was constructed by the Maximum likelihood (ML) method in MEGA12 software, with the Bootstrap value set at 1000. Subsequently, iTOL (https://itol.embl.de/ (accessed on 23 April 2023)) was used for visualization and beautification of the phylogenetic tree.

### 4.5. Gene Structure and Motif Analysis of Soybean Dof Members

The structure of soybean *Dof* genes, including cDNA sequences and the corresponding genomic DNA sequences, was extracted from the soybean genome annotation file. Finally, TBtools v2.310 was used for visualization analysis. Online MEME (http://www.OMIcsclass.com/article/67 (accessed on 19 May 2023)) was employed to analyze the motif structure of soybean Dof members, with the maximum number, minimum width, and maximum width of motifs set at 20, 6, and 50, respectively.

### 4.6. Analysis of Collinearity and KaKs of Soybean Dof Members

The genome sequences and annotation files of Arabidopsis (TAIR10.55) and rice (v7.0) were downloaded from the Phytozome v13 website (https://phytozome-next.jgi.doe.gov/ (accessed on 23 May 2023)). The genome sequences and annotation files of tobacco (Niben261) were downloaded from the Sol Genomics Network database (https://solgenomics.net/ (accessed on 23 May 2023)). The genome sequences and annotation files of potato (SolTub_3.0) were downloaded from the Ensemble Plants database (https://plants.ensembl.org/index.html (accessed on 23 May 2023)). The MCScanX program was used to analyze the collinearity of *Dof* members in soybean with those in Arabidopsis, rice, tobacco, and potato. The syntenic relationships within *Dof* members of soybean were visualized using the TBtools software. The non-synonymous/synonymous substitution rate (Ka/Ks) is an important indicator for measuring the selection pressure on gene evolution. TBtools software was used to visualize and analyze the Ka/Ks results (Ka/Ks < 1 indicates purifying selection, Ka/Ks = 1 indicates neutral evolution, and Ka/Ks > 1 indicates positive selection).

### 4.7. Prediction of Cis-Acting Elements of Soybean Dof Members

The 2000 bp sequence upstream of each soybean *Dof* member was downloaded from the Phytozome v13 website. The sequences were analyzed via the CARE search tool in the PlantCARE database (http://bioinformatics.psb.ugent.be/webtools/plantcare/html (accessed on 26 May 2023)). The *cis*-acting elements were visualized and summarized by the Basic Biosequence View and HeatMap modules in TBtools.

### 4.8. Plant Materials and Pathogen Strain

Jinong 4507 (JN4507) is a soybean variety susceptible to *P. sojae* infection. The high-yield variety Jinong 28 (JN28), developed by the Plant Biotechnology Center of the Jilin Agricultural University, is resistant to *P. sojae* infection. The special variety Dongnong 50 (DN50), developed by the College of Agriculture of the Northeast Agricultural University, is susceptible to *P. sojae* infection. In this study, JN28 was used for gene isolation, and DN50 was used for soybean transformation. Soybean seedlings were grown in a greenhouse with vermiculite as the growth medium. The seedlings were cultivated under a photoperiod of 16L/8D, 25 °C, and the relative humidity of 70%.

*P. sojae* race 1 (PSR01), a dominant race in Jilin Province, was kindly provided by Professor Shuzhen Zhang from Northeast Agricultural University. PSR01 was cultured on V8 juice agar in a polystyrene dish and activated by incubation at 22–25 °C.

### 4.9. Resistance Identification, RNA Extraction, and Transcriptome Sequencing

To examine the phenotypes of JN4507 and JN28 in response to *P. sojae* infection, we used a sterile scalpel to inoculate the agar plugs covered by mycelia (2 cm × 2 cm) on wounded hypocotyls of 14-day-old soybean seedlings. The inoculum was prepared from 7-day-old fungal cultures grown on V8 juice agar. We extracted total RNA from the inoculation point of soybean seedlings with Trizol reagent (Sangon Biotech, Shanghai, China). RNA quality was determined by the Qubit2.0 RNA test kit (Thermo-Life, Waltham, MA, USA). A high-quality RNA sample (OD260/280 of 1.8 to 2.2, OD260/230 ≥ 2.0, RIN ≥ 6.5, 28S:18S ≥ 1.0, >10 μg) was used to construct the sequencing library. The transcriptome sequencing work was carried out by Sangon Biotech.

### 4.10. Quantitative RT-PCR

One Step RT-PCR Kit (Code No. PCR-311, TOYOBO, Tokyo, Japan) was used for standard expression determination, and the specific primers for the target genes and housekeeping genes were designed with Primer 5 software. The reaction procedure was described as follows: 95 °C for 30 s; followed by 40 cycles of 95 °C for 5 s, 60 °C for 15 s, and 72 °C for 45 s. The relative expression levels were calculated by the 2^−ΔΔCT^ method. *GmActin* (Glyma.18G290800.1) was used as the internal control.

### 4.11. Primer Sequences Used in the Present Study

The specific primers used for all assays are listed in Table S3.

## 5. Conclusions

A total of 78 soybean *Dof* genes were identified and phylogenetically divided into 9 subfamilies. Gene structure analysis showed that all *GmDofs* contained 0 to 2 introns, and most of them did not have introns. Motif and conserved domain analysis showed that all GmDofs contained a common motif (motif-1) and a typical conserved C_2_-C_2_ domain. Investigations into CREs indicated the presence of various stress-responsive, plant growth and development, hormone-responsive, and light-responsive regulatory elements in the promoter region of *GmDofs*. Subsequently, RNA-seq and qRT-PCR results showed that *GmDof63* was specifically expressed at high levels after *P. sojae* infection. *GmDof63* was strongly induced by SA and ETH treatments. *GmDof63* enhanced resistance to *P. sojae* infection in *GmDof63*-overexpressing transgenic soybean seedlings. Furthermore, the expression levels of *PR* genes *PR1a*, *PR4*, *PR5a*, and *PR10* were significantly up-regulated in *GmDof63*-overexpressing transgenic soybean seedlings. Our study provides a basis for further research on the functions of soybean *Dof* family members in biotic stress tolerance.

## Data Availability

Data are contained within the article and Supplementary Materials.

## Supplementary Materials

The following supporting information can be downloaded at: https://www.mdpi.com/article/10.3390/plants14233621/s1, Table S1: Detailed information of all identified soybean Dof members; Table S2: Segmental duplication of *GmDofs* among *Glycine max* chromosomes; Table S3: Oligonucleotide primers used in this study; Table S4: Source data underlying the graphs presented in the main figures. Figure S1: Detection of the T0 and T2 transgenic plants with PAT/Bar LibertyLink strips. Figure S2: The AAAG or TTTC motif in the promoter sequence of *PR1a*, *PR4*, *PR5a*, and *PR10*.
