# Supplementary figures and images for: Genome-Wide Analysis of the Dof Gene Family in Soybean and Functional Identification of GmDof63 in Response to Phytophthora sojae Infection
Source: Plants (Basel). 2025 Nov 27;14(23):3621. doi: 10.3390/plants14233621 (PMC12693740; doi:10.3390/plants14233621)

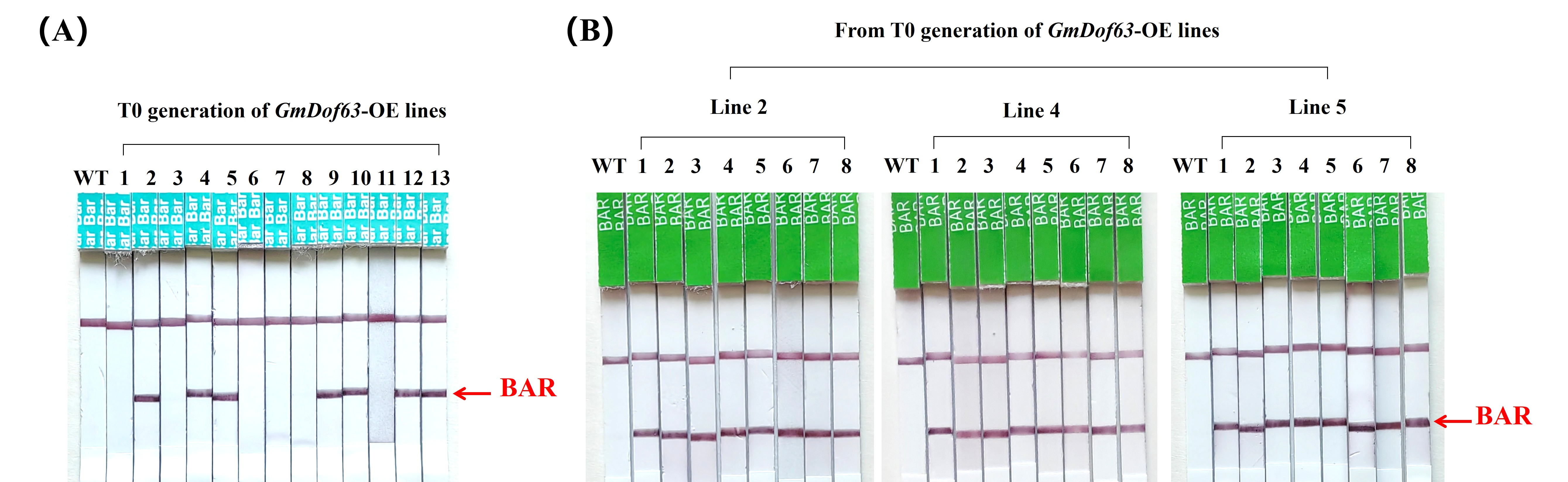

Supplement: Supplementary file 1 [file plants-14-03621-s001.zip › plants-3972908-supplementary/Figure S1.tif]

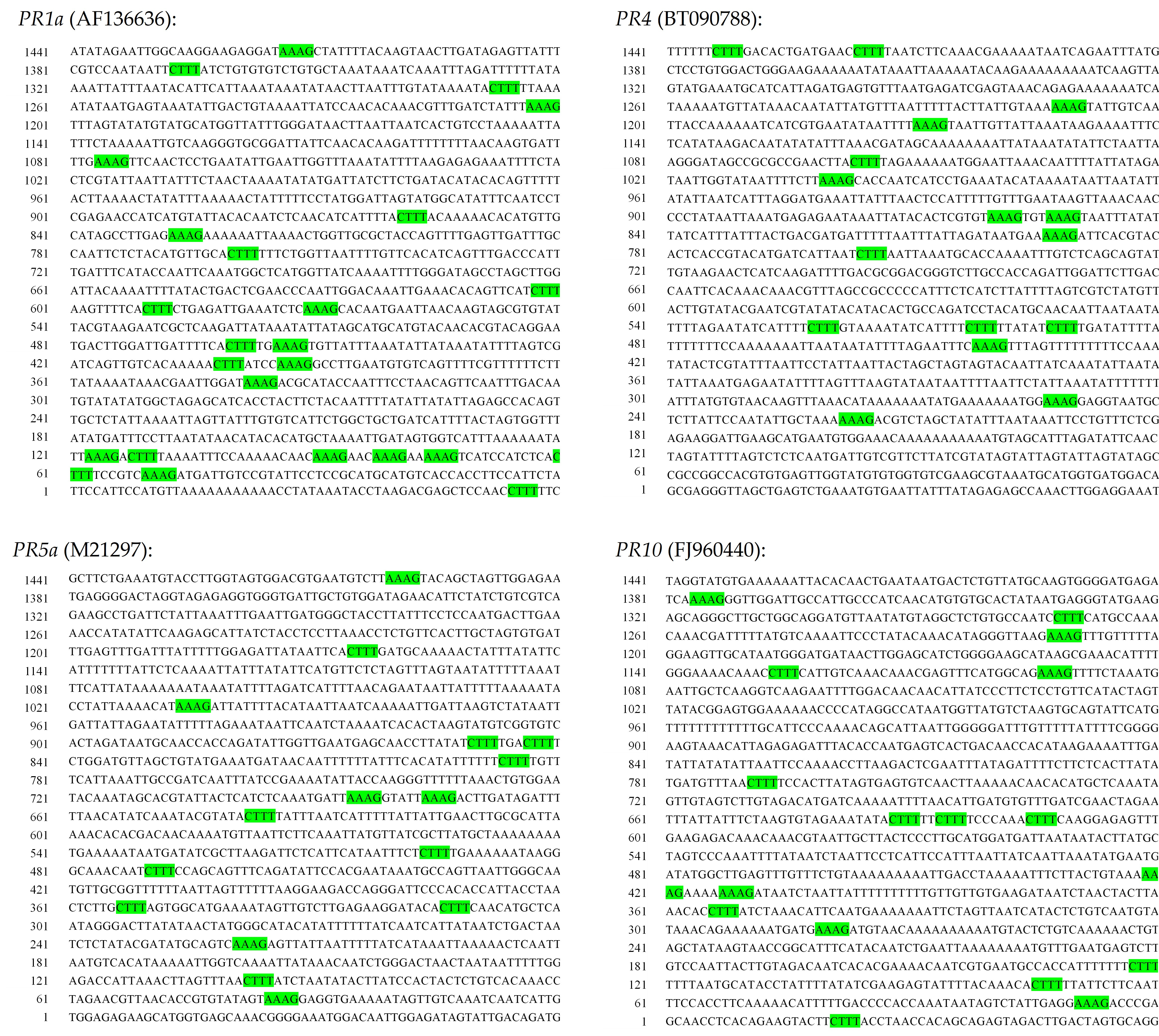

Supplement: Supplementary file 1 [file plants-14-03621-s001.zip › plants-3972908-supplementary/Figure S2.tif]
